# Beyond sperm competition: perspectives on sperm economy from external fertilizing fishes

**DOI:** 10.1098/rsos.261579

**Published:** 2026-11-04

**Authors:** Lisa Locatello, Maria B. Rasotto

**Affiliations:** ^1^ Department of Biology and Evolution of Marine Organisms, Stazione Zoologica Anton Dohrn, Fano Marine Center, Fano, Italy; ^2^ Department of Biology, University of Padua, Padua, Italy

**Keywords:** fishes, reproduction, behaviour, sperm economy, mating systems, reproductive investment, spermatogenesis

## Abstract

Research on sperm economy has traditionally focused on sperm competition as a major selective pressure shaping sperm production and allocation. In this review, we broaden this perspective by considering how other reproductive and ecological pressures influence the management of a costly and finite sperm supply. We synthesize evidence showing that in externally fertilizing fishes, high mating frequency, low female fecundity, prolonged spawning, rapid sperm dilution and costly male reproductive investment can favour sperm-economy strategies even when sperm competition is weak or absent. Across fish taxa, these constraints have led to convergent behavioural and physiological adaptations, including fractionated ejaculation, temporal matching of sperm release to egg deposition and manipulation of the fertilization microenvironment, which improve fertilization efficiency while minimizing sperm expenditure. We further highlight a recurring association between these strategies and semi-cystic spermatogenesis, consistent with the possibility that optimization of sperm use extends beyond ejaculate allocation to the regulation of sperm production during spermatogenesis. By integrating evidence from behaviour to reproductive physiology, this review reframes sperm economy as a general resource-allocation problem, with sperm competition representing one of several selective pressures. Externally fertilizing fishes emerge as an exceptional model for understanding the diverse evolutionary routes by which males optimize reproductive investment.

## 1. Introduction

Sperm production is not merely a background physiological process in reproduction but a central, and often limiting, component of male reproductive success. The long-standing view of sperm as an abundant and inexpensive commodity is increasingly untenable, as accumulating evidence over recent decades demonstrates that sperm production entails substantial energetic and physiological costs [1–3], and that sperm reserves can be significantly depleted over successive mating events [4–7]. Because sperm supply is inherently finite, males are expected to economize on both sperm production and usage to maximize fitness returns in accordance with the mating context [1,2,8,9].

### 1.1. Sperm economy under sperm competition: predictions and established patterns

Sperm competition, defined as competition between ejaculates of two or more males to fertilize the same set of ova [10], is traditionally regarded as the primary selective force shaping investment in sperm production and allocation. An extensive theoretical framework has been developed to explore the evolution of male sperm-economy strategies across gradients of sperm competition [2,11,12]. Under highly competitive conditions, males transferring larger quantities of sperm and sperm of higher quality are predicted to gain a numerical and functional advantage in fertilization success [13]. Consistent with these predictions, increases in relative testes size, and thus enhanced sperm production, as well as improvements in sperm quality under elevated levels of sperm competition represent taxonomically widespread patterns across both invertebrate and vertebrate taxa [14–17].

Moreover, males are expected to invest in strategies that reduce the risk of sperm competition, as well as to plastically adjust sperm production and allocation according to the reproductive value of individual mating events [8,9]. In fact, males limit sperm competition through a wide range of behavioural and physiological mechanisms, including pre- and post-copulatory mate guarding, behavioural exclusion of rivals and/or their sperm and ejaculate-mediated effects that lower female receptivity to subsequent matings and/or impair rival sperm performance [18–23]. In addition, males may respond dynamically to immediate competitive cues by fine-tuning their ejaculate investment. Such fine-scale and rapid adjustment includes accelerating the final stages of sperm maturation in response to heightened competition (‘sperm priming’) [24], the strategic modulation of sperm number and quality, as well as of seminal fluid amount and composition, according to the perceived sperm competition risk [2,9,19,25–28].

Since Nakatsuro & Kramer [4], studying a small characid fish, challenged the conventional view that sperm is virtually cost-free to produce, bony fishes, with their remarkable diversity in life histories, reproductive modes and mating systems, have become a major model taxon for studying sperm production and allocation. Fertilization mode is likely to influence the ecological and reproductive conditions under which sperm limitation arises. External fertilization exposes ejaculates to greater dilution and stochastic environmental effects, whereas internal fertilization reduces exposure to these sources of sperm loss but introduces other features, such as direct sperm transfer, sperm storage and temporal separation between mating and fertilization. However, when factors, such as sperm competition, female quality and mating frequency, constrain the probability of fertilization, both fertilization modes appear to converge on strategic solutions to economize sperm expenditure. Evidence includes an increased sperm number in response to sperm competition, a prudent sperm allocation in relation to female quality, as well as the balanced investment in pre-copulatory mate guarding and post-copulatory strategic sperm allocation under sperm competition [29–38]. Moreover, recent evidence in the dusky frillgoby, *Bathygobius fuscus*, suggests that male fish can also actively modify the fertilization environment to mitigate sperm competition by behaviourally displacing rival sneaker males’ sperm from the nest through tail-fanning, while compensating for the resulting sperm loss by increasing their own sperm release during spawning [39]. This strategy represents a striking parallel to sperm displacement mechanisms long recognized in internal fertilizers like insects [40].

## 2. Non-competitive selective pressures on sperm economy

Although research has largely emphasized post-copulatory sexual selection, and sperm competition in particular, as a major selective driver of ejaculate evolution, the energetic costs of sperm production and the inherently finite nature of sperm supply make ejaculate allocation a general resource-allocation problem. These constraints may generate strong selective pressures to regulate sperm production and expenditure across reproductive contexts, including those in which competition with rival ejaculates is weak or absent [41].

Optimal ejaculate investment may therefore be shaped not only by competition among sperm, but also by the need to balance sperm availability against mating rate, fecundity returns, spawning duration, fertilization efficiency and energetic investment in other reproductive activities (e.g. paternal care). In situations in which sustained high sperm production is not favoured for competitive purposes, allocating excess sperm yields little benefit; accordingly, selection is expected to favour reduced sperm production and/or increasingly precise sperm allocation, supported by behavioural, physiological and anatomical mechanisms that enable males to use their sperm supply efficiently.

Here, we broaden current views of sperm economics, focusing on externally fertilizing fishes which represent approximately 98% of all bony fishes and encompass the vast majority of the bewildering variability of reproductive modes present in this taxon. We reframe the underappreciated sources of selection acting on sperm production and allocation, and examine the strategies males employ to cope with biological and environmental constraints independently of sperm competition.

### 2.1. High mating frequency and sperm depletion

High mating frequency can impose substantial demands on male sperm reserves when the rate of sperm expenditure exceeds that of sperm replenishment. This constraint is particularly evident in many coral reef fishes, where males engage in multiple mating events within a single day, such that high mating rates alone can constrain sperm availability [41,42]. Consequently, males may be unable to produce sufficient sperm to achieve maximal fertilization across all spawning events, thereby limiting paternity even in the absence of rival ejaculates and generating strong selective pressure for efficient sperm usage. Compelling evidence for sperm-economy strategies under high mating rates comes from the bluehead wrasse *Thalassoma bifasciatum*. In this species, males spawn either in groups or in pairs. During group spawning, where sperm competition is intense, males release approximately six times more sperm than during pair-spawning events, which occur in the absence of sperm competition [29], demonstrating that sperm competition strongly influences ejaculate allocation. Importantly, however, sperm economization is also evident within the non-competitive context of pair spawning. Indeed, pair-spawning males with exceptionally high mating success (exceeding 50 matings per day) reduce sperm output per mating, resulting in fertilization rates approximately 5% lower than those of males with more moderate daily mating rates [31]. By conserving sperm per spawn, highly successful males are able both to continue defending females and to maintain relatively high fertilization success across successive spawning events [31,41]. Thus, while sperm competition clearly affects sperm allocation in this species, the adjustment of sperm expenditure among pair-spawning males demonstrates that high mating frequency can independently generate selection for sperm economy.

### 2.2. Female fecundity and reproductive value of mating events

When sperm supplies are limited, allocating the same amount of sperm to every mating event may be inefficient if females differ in fecundity. Males are therefore expected to adjust sperm expenditure according to female fecundity, allocating more sperm to mating events offering greater potential fertilization returns. Evidence for such adjustment in the absence of immediate sperm competition comes, again, from *T. bifasciatum*. During pair spawning, sperm release increases with female body size and clutch size [29,32], indicating that males fine-tune sperm expenditure according to the potential number of eggs available for fertilization. By adjusting sperm release in relation to female fecundity, males increase the probability of fertilizing a substantial proportion of the egg clutch while avoiding unnecessary sperm expenditure. The mechanistic basis of this precise control has been elucidated through detailed analyses of the morphology and histological structure of the male genital papilla, sperm duct and associated musculature [43].

A similar pattern of sperm economy relative to female size and clutch size is observed in the bucktooth parrotfish *Sparisoma radians*, a species suffering a low risk of sperm competition and in which strategic sperm allocation maintains an adequate daily standing crop to support multiple spawning events without a decline in sperm release as the day progresses [32]. Sperm-economy strategies are also evident in the Mediterranean pearly razorfish *Xyrichthys novacula*, a harem-holding fish in which males defend territories containing variable numbers of females and sperm competition has not been reported. In this species, spawning rates are substantially lower than in *T. bifasciatum* and *S. radians*, suggesting weaker selective pressure for sperm economy, and accordingly sperm release per spawn is not related to female size [30]. Nonetheless, *X. novacula* also clearly operates under a regime of sperm economy: males holding larger harems release fewer sperm per spawn, yet the male with the largest harem achieves the highest total daily number of fertilized eggs owing to access to more females. This strategy maximizes overall male reproductive output despite reduced per-spawn fertilization success [30].

Similar strategies of fractioning sperm expenditure in relation to female biology are also observed in combtooth blennies, a family of demersal-spawning fishes in which females parcel their egg batches among different males and males partition their sperm load into several successive ejaculations during mating, thereby adjusting sperm release to the duration of egg deposition [44]. Blenny species experiencing higher risk of sperm competition produce greater numbers of sperm, whereas smaller species in which sperm competition does not occur and female fecundity is low, exhibit reduced sperm expenditure [44]. These patterns are consistent with the prediction that, in contexts where the risk of sperm competition is low or absent, and males experience low fecundity returns while incurring high reproductive costs, often associated with substantial investment in paternal care, the prudent allocation of smaller ejaculates is expected.

Patterns of sperm allocation relative to the fecundity return extend beyond individual species and are evident at a macroevolutionary scale, as demonstrated by comparative analyses across fishes [45,46]. Together, these findings underscore the pivotal role of female reproductive biology in shaping male sperm investment, independently of, and in addition to, sperm competition.

### 2.3. Temporal constraints imposed by prolonged spawning

Like blennies, many benthic fishes that release demersal eggs on sand, rocks, vegetation or inside nests are characterized by prolonged spawning durations, which can further influence ejaculate allocation. In gobies and batrachoids, for example, egg laying often lasts several hours, during which males must simultaneously invest in sustained sperm release and in defending the nest [47–52]. In these species, rather than releasing sperm freely into the water, territorial males partition their ejaculate into viscous sperm trails that adhere to the nest roof. The substantial mucous component of the seminal fluid regulates the timing of sperm release from these trails into the surrounding water: as females slowly attach eggs sequentially to the nest roof, sperm are gradually released, enabling fertilization of eggs laid over several hours [53–55]. This mechanism constitutes an efficient sperm-economy strategy, ensuring fertilization throughout an extended spawning event while minimizing unnecessary sperm expenditure.

In gobies, sperm release may also strategically begin before female egg laying [55], a pre-emptive strategy that ensures sperm are already present and active at the fertilization site when eggs are released. Such early release likely mitigates environmental constraints typical of external fertilization, where precise timing and gamete spatial proximity are critical. A comparable strategy occurs in bitterlings, where females deposit eggs deep within freshwater mussels using an elongated ovipositor, and fertilization takes place inside the gill chamber as sperm are drawn in with the inhalant water current [56–58]. By releasing sperm embedded in mucous seminal fluid over the mussel's inhalant siphon prior to egg deposition, males ensure that viable sperm are already present inside the gill chamber when eggs arrive, thereby gaining sperm precedence and increasing fertilization success [57].

### 2.4. Dilution constraints imposed by external fertilization

Beyond ensuring continuous fertilization during prolonged spawning events, the strategic release of sperm embedded in viscous seminal fluid may represent a general adaptation to counteract rapid sperm dilution, a major challenge in externally fertilizing fishes that can strongly drive ejaculate evolution [46]. In the small red scorpionfish *Scorpaena notata*, a species characterized by low female fecundity, eggs are released in a floating gelatinous mass, while sperm are embedded in an abundant, viscous seminal fluid [59,60]. This mode of sperm release enhances sperm retention in close proximity to the eggs, facilitates fertilization of the entire gelatinous egg mass, and minimizes sperm dispersion [59,60].

An extreme behavioural strategy aimed at maximizing contact between sperm and eggs and counteracting dilution is found in members of the family Callichthyidae, the armoured catfishes, which employ sperm drinking. In these fishes, most of which have very low female fecundity, with fewer than 200 eggs released per spawn, females ingest sperm by attaching their mouth to the male's anal region during courtship [61]. The sperm subsequently pass through the gastrointestinal tract and are released from the female's urogenital pore and reach the area between the pelvic fins, where ovulated eggs are temporarily stored and fertilized [61]. Among callichthyids, *Corydoras* species exhibiting sperm drinking possess well-developed seminal vesicles that produce highly viscous seminal fluid, whereas species that do not engage in sperm drinking lack such specialized accessory organs [62]. Recent proteomic analyses of seminal fluid in *Corydoras aeneus* further reveal that seminal proteins suppress sperm motility during passage through the digestive tract, allowing sperm to become motile only when released near the eggs [63]. This mechanism maximizes sperm economy and fertilization efficiency by tightly controlling the spatial and temporal activation of sperm [63]. Optimal egg fertilization is ensured inside the female mouth in the monogamous, mouth-brooding cichlid *Eretmodus cyanostictus* [64]. In the mandarinfish *Synchiropus splendidus*, a promiscuous pair-spawner characterized by low female fecundity, relatively small ejaculate size per mating [65], and high mating rate in more successful males, close contact between gametes reduces sperm waste [66]. During spawning rises, the male's anal fin closely frames the female genital opening, effectively funnelling the simultaneous release of eggs and sperm and enhancing fertilization efficiency (figure 1a). Close vicinity during gamete release is also observed in *Solea senegalensis*, another pair-spawner characterized by low female fecundity, and in which the mating couple during spawning swim to the surface and hold the genital openings closely together during gamete release [67,68].

**Figure 1.**
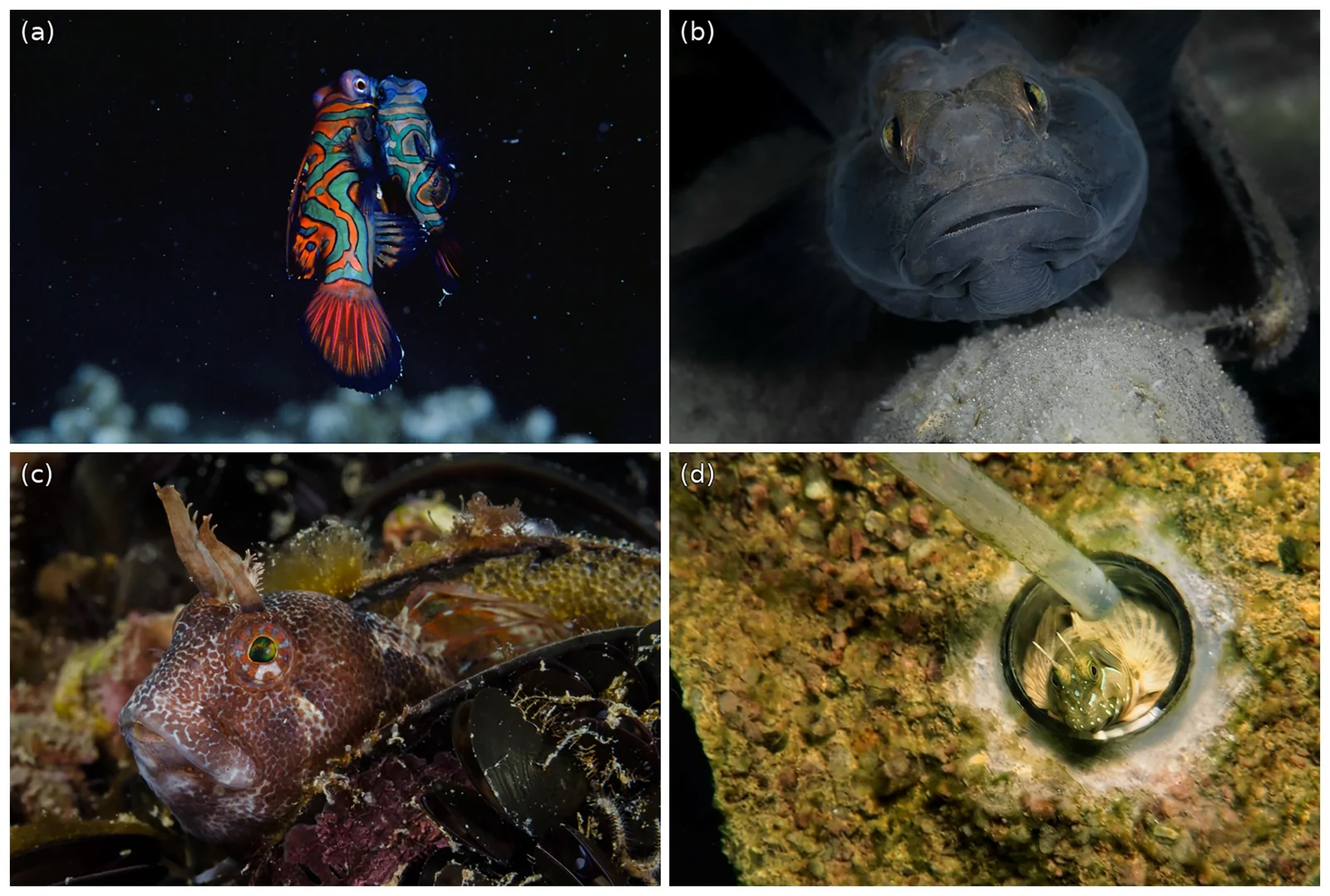
Behavioural adaptations and nest characteristics that reduce sperm dispersion in external fertilizing fishes. (a) Spawning pair of the mandarin fish *Synchiropus splendidus*, in which the male closely apposes his anal fin to the female's genital opening during spawning rises, funnelling the simultaneous release of eggs and sperm (photograph © George Mitcheson, used with permission). (b) *Gobius niger* male guarding a nest under a narrow rock crevice, where spawning occurs in a confined space that limits sperm dispersal (photograph © Marco Boncompagni, used with permission). (c) Tentacled blenny, *Parablennius tentacularis*, male nesting and spawning inside an empty mollusc shell, which provides a sheltered cavity for gamete release (photograph © Marco Boncompagni, used with permission). (d) *Aidablennius sphynx* spawning inside an artificial nest while sealing the entrance with its pectoral fins, thereby restricting water flow and minimizing sperm loss during fertilization.

In species where sperm competition is uncommon, such as monogamous gobies, jawfish and several blennies, sperm dispersion is nonetheless minimized through the use of small nests (holes, crevices and empty bivalves) or burrows that constrain water flow and are often actively sealed by males before mating, or blocked with pectoral fins during mating (figure 1b–d) [69–76]. By reducing sperm loss from the fertilization site, this behavioural control of the spawning microenvironment enhances fertilization efficiency and represents a key sperm-economy strategy. Fertilization and egg development are similarly secured in the spotted snakehead, *Channa punctata*, through the construction of a unique and complex nest system consisting of tunnels dug through vegetation that lead into a central nest chamber, creating interconnected pathways and enclosed spaces [47,77]. Protected spots are also available to clingfishes that lay their adhesive eggs in a single layer on the underside of rocks or cobbles in intertidal zones or tide pools [47,78,79].

## 3. Economy of sperm production via semi-cystic spermatogenesis

Most of the fish species examined so far, in which sperm competition is weak or absent, but males nonetheless employ physiological and behavioural strategies to allocate sperm efficiently, also exhibit a semi-cystic type of spermatogenesis. This recurring association raises the possibility that sperm economy extends beyond ejaculate allocation to mechanisms of ejaculate production (table 1 and references therein). In these species, spermatids rather than spermatozoa are released from the testis lobules and subsequently complete maturation along the reproductive duct system before reaching the genital opening [57,71,89,111,132]. The level of semi-cystic spermatogenesis varies among species, ranging from high, when spermatids are released at early stages of maturation, to low, when spermatids are released at the late stage of differentiation to sperm [44,132]. The major consequence of this process is the asynchronous maturation of spermatids, which reduces the number of sperm maturing simultaneously. Accordingly, species exhibiting a high level of semi-cystic spermatogenesis exhibit a more developed duct system and produce smaller ejaculates than species with a low level of semi-cystic spermatogenesis [44]. As detailed in table 1, most of the species in which semi-cystic spermatogenesis occurs, despite belonging to diverse families, are characterized by monogamous and/or pair-spawning mating systems, in which males face little or no risk of sperm competition, invest heavily in paternal care (e.g. mouth-brooding, pouch brooding, elaborate nest construction and prolonged care), and consequently incur high reproductive costs while experiencing low fecundity returns per mating owing to the release of relatively small numbers of eggs by females. Sperm competition, when present, may affect the level of semici-cystic spermatogenesis and, consequently, sperm production. Indeed, among blennies, the territorial nesting males of *Salaria pavo* and *Scartella cristata*, two species exhibiting alternative male mating tactics [117,118], show a lower level of semi-cystic spermatogenesis and release sperm-rich ejaculates [44]. By contrast, sneaker males, which consistently mate under sperm competition, have larger testes than nesting males, while their duct system is poorly developed or absent, suggesting the occurrence of cystic spermatogenesis [117,118]. Similarly, sneaker males of the triplefins *Tripterygion tripteronotus* and *T. delaisi* exhibit a much less developed testicular gland than territorial males [133].

**Table 1.** Reproductive characteristics associated with semi-cystic spermatogenesis in teleost fishes. The table summarizes available information on mating system, parental care, fertilization dynamics, female fecundity, sperm production and allocation of species exhibiting semi-cystic spermatogenesis. It also includes species belonging to the same families with similar reproductive characteristics, but for which the type of spermatogenesis has not yet been investigated, as well as species known to exhibit semi-cystic spermatogenesis for which reproductive information remains incomplete. Fecundity and sperm traits are reported using the metrics provided in the original studies, as differences in methodology and reporting often preclude their reliable standardization. Empty cells indicate that the corresponding information was not available in the surveyed literature, whereas n.a. indicates that the type of spermatogenesis has not yet been investigated. When retrieved from the original reference, SE indicates the standard error of the mean. When the original reference reported SD and the corresponding sample size was available, SD was converted to SE. Data marked with an asterisk (*) were obtained by digitizing published figures using WebPlotDigitizer 5.2. The table summarizes the available evidence, but no formal comparative analyses were performed on it. Accordingly, any association between sperm economy strategies and semi-cystic spermatogenesis should be considered descriptive rather than supported by formal quantitative analysis.

family/species	mating system	parental care	fertilization dynamics	no. of eggs/spawn	fecundity	sperm production/stripping	sperm allocation/spawn	type of spermatogenesis	references
Callichthyidae:									
*Callichthys callichthys*		male caring for eggs in floating bubble nest in cavities of tree roots		up to 120	mean 1599 ± 191 SE (batch fecundity)			semi-cystic	[62,80,81]
*Hoplisoma flaveolus*		no						semi-cystic	[82]
*Hoplisoma bondi*		no	sperm drinking	about 100				semi-cystic	[62,83]
*Hoplisoma ehrhardti*		no	sperm drinking					semi-cystic	[62]
*Hoplisoma potaroense*		no	sperm drinking					semi-cystic	[62]
*Hoplosternum littorale*	polygynous	male caring for eggs in floating bubble nest on leaves	sperm drinking	6000–9000	mean 5509 ± 868 SE (batch fecundity)			semi-cystic	[62,84]
*Megalechis thoracata*	polygynous	male caring for eggs in floating bubble nest on leaves			mean 2566 ± 284 SE (batch fecundity)			n.a.	[81]
*Osteogaster aenea*	promiscuous	no	sperm drinking	about 30				semi-cystic	[61,62]
Ariidae:									
*Genidens genidens*		male mouthbrooder			up to 30 (batch fecundity)			semi-cystic	[85,86]
Ictaluridae:									
*Ameiurus nebulosus*	monogamous	paternal or biparental care of eggs in nest, occasionally female care			mean 3227 ± 387.7 SE range 334–11471 (*n* = 52)(absolute fecundity)			semi-cystic	[87]
Ophidiidae:									
*Ophidion barbatum*		male caring gelatinous mass of eggs						semi-cystic	[88]
Syngnathidae:									
*Syngnathus abaster*	polygamous	male brood pouch		mean 24.19 ± 1.29 SE range 10–32 (*n* = 21) (TOT eggs from different females)				semi-cystic	[89–91]
*Syngnathus typhle*	promiscuous	male brood pouch		mean 44.8 ± 2.32 range 23–65 (*n* = 30) (TOT embryos from different females)				semi-cystic	[89,92,93]
*Syngnathus acus*	polygamous	male brood pouch		mean 34 ± 2.65 SE range 2–81 (*n* = 11) (embryos)				semi-cystic	[89,90,94,95]
*Hippocampus guttulatus*	monogamous	male brood pouch	fertilization inside the male brood pouch	mean 213 ± 10.25 SE (*n* = 117)				semi-cystic	[96–101]
*Hippocampus kuda*		male brood pouch	fertilization inside the male brood pouch			mean 304 ± 77 SE (*n* = 4)		n.a.	[47,102]
Scorpaenidae:									
*Scorpaena porcus*		no (eggs released in floating gelatinous mass)			range absolute (annual): 5245–20602 range relative (g / body mass): 69–163 (*n* = 10)			semi-cystic	[47,103,104]
*Scorpaena scrofa*		no (eggs released in floating gelatinous mass)						semi-cystic	[47,103]
*Scorpaena notata*		no (eggs released in floating gelatinous mass)	sperm in abundant viscous seminal fluid		range absolute (annual): 6000–33000 range relative (g / body mass): 69.5–396.6 (*n* = 45)			semi-cystic	[59,60]
Gymnotidae:									
*Gymnotus carapo*		male mouthbrooder		about 200–247				semi-cystic	[105,106]
Soleidae:									
*Solea senegalensis*	promiscuous/pair spawning	no	close alignment of the genital openings during ascendance			range 40–60 × 10^6^ (*n* = 11)		semi-cystic	[68,107,108]
Channidae:									
*Channa punctata*		biparental care in elaborate tunnel nest through vegetation			range 2300–29600 (absolute fecundity)			semi-cystic	[47,109,110]
Callionimidae:									
*Synchiropus splendidus*	promiscuous/pair spawning	no	male anal fin funnel around female genital pore	range 12–205 (*n* = 35)			51.8–235 × 10^3^ (*n* = 22)	semi-cystic	[65, Rasotto & Sadovy unpublished data]
Gobioesocidae:									
*Lepadogaster lepadogaster*		male caring for eggs under stones		200–250				semi-cystic	[47,111]
Opistognathidae:									
*Opistognathus whitehurstii*		male mouthbrooder						semi-cystic	[112,113]
Blenniidae:									
*Aidablennius sphynx*	promiscuous	male caring for eggs in small hole	inside nest	up to 784			mean 1434.55 ± 146.61 SE × 10^3^ (*n* = 21)	semi-cystic	[44,73,114]
*Lipophrys adriaticus*	promiscuous	male caring for eggs in small hole	inside nest				mean 1631.65 ± 220.62 SE × 10^3^ (*n* = 15)	semi-cystic	[44,75,115]
*Lipophrys dalmatinus*	promiscuous	male caring for eggs in small hole	inside nest				mean 327.12 ± 47.16 SE × 10^3^ (*n* = 10)	semi-cystic	[44,75,115]
*Lipophrys canevae*	promiscuous	male caring for eggs in small hole	inside nest					semi-cystic	[44,75,115]
*Parablennius tentacularis*	promiscuous	male caring for eggs in small hole/empty mussel	inside nest	mean 8459.31 ± 722.97 SE range 2817.5–18693.6 (*n* = 23) (TOT from different females)			mean 1258.47 ± 310.14 SE × 10^3^ (*n* = 5)	semi-cystic	[44,115,116]
*Parablennius incognitus*	promiscuous	male caring for eggs in small hole/empty mussel	inside nest					semi-cystic	[75,115]
*Salaria pavo*	promiscuous	male caring for eggs in small hole/empty mussel	inside nest				mean 10735.68 ± 1685.38 SE x 10^3^ (territorial males; *n* = 13)	semi-cystic	[44,117]
*Scartella cristata*	promiscuous	male caring for eggs in small hole	inside nest				mean 6589.30 ± 507.05 SE × 10^3^ (territorial males; *n* = 11)	semi-cystic	[44,118]
*Coryphoblennius galerita*	promiscuous	male caring for eggs in small hole	inside nest					semi-cystic	[115,119]
Tripterygiidae:									
*Tripterygion tripteronotum*	promiscuous	male caring for eggs attached to algae						semi-cystic	[47,120–122]
*Tripterygion delaisi*	promiscuous	male caring for eggs attached to algae						semi-cystic	[47,120–122]
*Enneanectes boehlkei*		male caring for eggs attached to algae						semi-cystic	[120–122]
Labrisomidae:									
*Labrisomus nuchipinnis*	promiscuous	male caring for eggs attached to algae						semi-cystic	[120–122]
*Labrisomus haitiensis*	promiscuous	male caring for eggs attached to algae						semi-cystic	[121–123]
*Labrisomus bucciferus*	promiscuous	male caring for eggs attached to algae						semi-cystic	[120–122]
*Labrisomus xanti*	promiscuous	male caring for eggs attached to algae						semi-cystic	[120–122]
*Paraclinus nigripinnis*	promiscuous	male caring for eggs in rocky hole/empty mussel						semi-cystic	[120–122]
*Malacoctenus versicolor*	promiscuous	male caring for eggs in rocky hole/empty mussel						semi-cystic	[120–122]
*Malacoctenus triangulatus*	promiscuous	male caring for eggs in rocky crevice/hole						semi-cystic	[120–122]
*Malacoctenus macrops*	promiscuous	male caring for eggs in rocky crevice/hole						semi-cystic	[120–122]
*Paraclinus sini*	promiscuous	male caring for eggs in cavity/hollow						semi-cystic	[120–122]
Clinidae:									
*Clinitrachus argentatus*	promiscuous	male caring for eggs attached to vegetation						semi-cystic	[47,121,122]
Dactiloscopidae:									
*Platygillellus rubrocinctus*		spawning in sand/gravel; male care						semi-cystic	[121]
									
Gobiidae:									
*Amblygobius nocturnus*	monogamous	male caring for eggs in multichambered burrow						semi-cystic	[71]
*Amblygobius phalaena*	monogamous	male caring for eggs in burrow	inside nest	range 37665–38443				n.a.	[123]
*Valenciennea muralis*	monogamous	male caring for eggs in burrow						semi-cystic	[71]
*Signigobius biocellatus*	monogamous	male caring for eggs in sealed nest						n.a.	[69]
*Paragobiodon echinocephalus*	monogamous	male builds a nest by cropping the coral tissue from a branch		mean 792 ± 51.4 SE; range 204–1629 (*n* = 44)				n.a.	[124]
*Pomatoschistus marmoratus*	promiscuous	male caring for eggs in empty mussel	sperm in mucous trail	mean 1159 ± 87.5 SE range 614–2271 (*n* = 43)				n.a.	[72]
*Knipowitschia panizzae*	promiscuous	male caring for eggs in empty mussel	sperm in mucous trail	mean 253 ± 14 SE; range 107–371 (*n* = 26)			range 1.8–7.1 × 10^3^ ml^–1^	n.a.	[53,74]
Acheilognathidae:									
*Rhodeus amarus*	promiscuous	eggs protected inside mussel; no direct cares	sperm in viscous seminal fluid; fertilization inside mussel	mean 2.9 ± 0.2 SE		mean 7103.4 ± 852 SE × 10^5^ (*n* = 25) (*)		semi-cystic	[47,57,125]
Macrouridae:									
*Nezumia aequalis*		no						semi-cystic	[126]
*Coelorinchus mediterraneus*		no						semi-cystic	[126]
Lophiidae:									
*Lophiomus setigerus*		no			range 3.3 × 10^5^–6.1 × 10^5^ (*n* = 20) (batch fecundity)			semi-cystic	[127,128]
*Lophiomus litulon*		no			range 0.31 × 10^6^–1.54 × 10^6^ (*n* = 15)				[129]
Notopteridae:									
*Notopterus notopterus*		male caring for eggs laid on stones or submerged vegetation						semi-cystic	[130]
Characidae:									
*Bryconops affinis*		no						semi-cystic	[131]

Non-competitive constraints on sperm production may also favour the occurrence of semi-cystic spermatogenesis in species lacking parental care. For instance, in the European bitterling, *Rhodeus amarus*, the high ejaculation rate (approx. 250 ejaculations per day), the associated risk of sperm depletion, exacerbated by sperm competition, and the small number of eggs released per batch (2–3) are thought to drive the production of numerous, small ejaculates [7,57]. Moreover, because semi-cystic spermatogenesis appears to influence the amount and consistency of seminal fluid, it may also contribute to counteracting sperm dilution. In the small red scorpionfish *Scorpaena notata*, the production of a viscous ejaculate, which facilitates sperm adhesion to the gelatinous egg mass, has been proposed to result from the premature opening of the spermatogenetic cysts [59]. This early cyst opening may allow the Sertoli cells, surrounding the cysts, to secrete an abundant, gelatinous seminal fluid [59].

Semi-cystic spermatogenesis may therefore represent a mechanism that allows externally fertilizing fishes experiencing low or no sperm competition to limit sperm production and minimize sperm wastage.

## 4. Conclusions and perspectives

This overview corroborates recent evidence challenging the long-standing assumption that sperm economy is primarily a consequence of sperm competition [46,134]. In external fertilizing fishes, patterns of ejaculate investment, indeed, appear to be influenced by a set of constraints unrelated to competition between ejaculates. High mating frequency, low female fecundity, prolonged spawning duration, rapid sperm dilution and substantial male reproductive investment may all constrain sperm availability and favour efficient sperm use even when competition between ejaculates is weak or absent. From this perspective, sperm economy can be viewed as a general resource-allocation problem, in which multiple selective pressures, including but not limited to sperm competition, shape how males optimize the production and expenditure of a costly and finite sperm supply.

Across a wide taxonomic range, fishes have evolved a remarkable diversity of behavioural and physiological strategies that converge on a common functional goal: maximizing fertilization efficiency while minimizing sperm expenditure. These strategies include fractionated ejaculation, temporal matching of sperm release to egg deposition, manipulation of the fertilization microenvironment, extreme behaviours that maximize gamete contact and seminal fluid adaptations that counteract sperm dilution. Importantly, many of these mechanisms operate independently of sperm competition and are instead tightly linked to female reproductive biology, mating system and ecological context (table 1). Crucially, this convergence extends beyond ejaculate allocation to the level of sperm production itself. Species exhibiting these sperm-economy strategies in the absence of sperm competition often exhibit a semi-cystic mode of spermatogenesis, which may facilitate economization of sperm production through asynchronous maturation of spermatids (table 1). This association is consistent with the hypothesis that sperm economy is not achieved solely at the moment of ejaculation or fertilization but also through the regulation of sperm production during spermatogenesis.

Our survey (table 1) also highlights species belonging to families in which semi-cystic spermatogenesis occurs, and sharing one or more reproductive traits associated with this type of spermatogenesis, but for which the type of spermatogenesis remains unknown. These species may therefore represent plausible candidates for the occurrence of semi-cystic spermatogenesis. They include the hoplo armoured catfish *Megalechis thoracata*, the spotted seahorse *Hippocampus kuda*, the monogamous gobies *Amblygobius phalaena*, *Signigobius biocellatus* and *Paragobiodon echinocephalus*, the Adriatic dwarf goby *Knipowitschia panizzae* and the marbled goby *Pomatoschistus marmoratus*. Several other external fertilizers, not included in our survey because of the limited information on both sperm production and allocation, may also represent potential candidates for semi-cystic spermatogenesis. These include, for instance, cichlids, tilefishes, cardinalfishes and clownfishes, in which monogamous mating systems and parental care are common [135]. Conversely, table 1 also includes species known to exhibit semi-cystic spermatogenesis, but whose reproductive biology remains poorly understood, such as the deep-sea fishes *Nezumia aequalis*, *Coelorinchus mediterraneus* and *Lophiomus setigerus*. Detailed information on spawning frequency, fertility, the occurrence of sperm competition and sperm production in these and other taxa will provide valuable opportunities to formally test, by comparative analysis, the proposed association between sperm economy strategies and semi-cystic spermatogenesis.

Based on currently available information, this mode of spermatogenesis has so far been reported only in bony fishes. However, suppression of the final stages of spermiogenesis has been observed in the Eurasian bullfinch, *Pyrrhula pyrrhula*, a passerine bird whose sperm retain a spermatid-like morphology [136]. In this species, sperm competition is absent or rare, and the peculiar mode of spermatogenesis has been suggested to represent an energy-saving response to the reduced risk of sperm competition [136]. More broadly, comparative studies in mammals and birds indicate that variation in testicular traits associated with mating systems and post-copulatory sexual selection extends beyond testis size and overall sperm output to encompass testicular architecture, spermatogenic efficiency and rates of spermatogenesis [137–143].

The resource constraints underlying sperm economy are not specific to external fertilization. Thus, the evolutionary principle emerging from this review may extend beyond the specific mechanisms documented in externally fertilizing fishes: selective pressures associated with female fecundity, mating system and mating frequency, may shape sperm economy at multiple stages, from sperm production and maturation to sperm allocation, regardless of fertilization mode. By shifting the focus from sperm competition to constraint-driven sperm economy, this synthesis provides a broader and more unified framework for understanding ejaculate evolution. It highlights externally fertilizing fishes as exceptional models for testing how ecological and reproductive constraints shape the evolution of sperm production and allocation, and generates testable hypotheses about the evolutionary links among mating systems and mating dynamics, fertilization biology and sperm-economy strategies.

## Data Availability

This article has no additional data.
